# Prevalence of River Epilepsy in the Orientale Province in the Democratic Republic of the Congo

**DOI:** 10.1371/journal.pntd.0004478

**Published:** 2016-05-03

**Authors:** Robert Colebunders, Floribert Tepage, Ente Rood, Michel Mandro, Emmanuel Nji Abatih, Gisele Musinya, Germain Mambandu, José Kabeya, Michel Komba, Bethany Levick, John L Mokili, Anne Laudisoit

**Affiliations:** 1 Global Health Institute, University of Antwerp, Antwerp, Belgium; 2 National Onchocerciasis Control Program, Kisangani, Democratic Republic of the Congo; 3 Royal Tropical Institute, Amsterdam, The Netherlands; 4 Provincial Health Division Ituri, Bunia, Democratic Republic of the Congo; 5 Department of Biomedical Science, Institute of Tropical Medicine, Antwerp, Belgium; 6 Medical Doctor, Bunia, Democratic Republic of the Congo; 7 Provincial Ministry of Public Health, Kisangani, Democratic Republic of the Congo; 8 Biodiversity Monitoring Centre (CSB), Faculty of Sciences, University of Kisangani, Kisangani, Democratic Republic of the Congo; 9 Institute of Integrative Biology, School of Biological Sciences, University of Liverpool, Liverpool, United Kingdom; 10 Biology Department, San Diego State University, San Diego, California, United States of America; 11 Evolutionary Biology group, University of Antwerp, Antwerp, Belgium; London School of Hygiene and Tropical Medicine, UNITED KINGDOM

## Abstract

**Background:**

An increased prevalence of epilepsy has been reported in many onchocerciasis endemic areas.

**Objective:**

To determine the prevalence and distribution of epilepsy in an onchocerciasis endemic region in the Democratic Republic of the Congo (DRC).

**Design/Methods:**

An epilepsy prevalence study was carried out in 2014, in two localities of the Bas-Uélé district, an onchocerciasis endemic region in the Orientale Province of the DRC. Risk factors for epilepsy were identified using a random effects logistic regression model and the distribution of epilepsy cases was investigated using the Moran’s I statistic of spatial auto-correlation.

**Results:**

Among the 12,776 individuals of Dingila, 373 (2.9%) individuals with epilepsy were identified. In a house-to-house survey in Titule, 68 (2.3%) of the 2,908 people who participated in the survey were found to present episodes of epilepsy. Epilepsy showed a marked spatial pattern with clustering of cases occurring within and between adjacent households. Individual risk of epilepsy was found to be associated with living close to the nearest fast flowing river where blackflies (Diptera: *Simuliidae*)–the vector of *Onchocerca volvulus*–oviposit and breed.

**Conclusions:**

The prevalence of epilepsy in villages in the Bas-Uélé district in the DRC was higher than in non-onchocerciasis endemic regions in Africa. Living close to a blackflies infested river was found to be a risk factor for epilepsy.

## Author Summary

An increased prevalence of epilepsy has been reported in many onchocerciasis (river blindness) endemic areas. In 2014, an epilepsy prevalence study was conducted in Dingila and Titule, two localities within the onchocerciasis endemic Orientale province of the Democratic Republic of Congo, both within the Bas-Uélé district. The prevalence of epilepsy was 2.9% in Dingila, and 2.3% in Titule. This is much higher than the epilepsy prevalence in non-onchocerciasis endemic regions in Africa. Epilepsy cases were clustered within and between adjacent households. Living close to a river infested with blackflies (the main vector of *Onchocerca* microfilariae) was found to be a risk factor for epilepsy.

## Introduction

An association between onchocerciasis and epilepsy was suspected as early as the 1930’s in Mexico [1] and later reports were published showing clustering of epilepsy in several African onchocerciasis foci [2–7]. Ecological studies carried out in onchocerciasis endemic areas in West, Central and East Africa found a strong association between the prevalence of onchocerciasis and of epilepsy [8, 9]. In previous case-control studies this association was less clear, but this was probably due to shortcomings in study design and the selection of comparison groups [9,10]. Moreover there seems to be an association between epilepsy and the degree of infection with *Onchocerca volvulus*. Indeed, in a study in Cameroon, performed before the introduction of annual ivermectin treatment (to control Onchocerciasis), the prevalence of epilepsy and the community microfilarial load were closely related [11]. Moreover a case-control study demonstrated that the microfilarial loads (microfilariae per skin snip) in the epileptic group were significantly higher than in the control group [11].

Nodding syndrome, a neurological syndrome of unknown origin is characterized by episodes of atonic seizures. It has been observed in Tanzania (Mahenge), South Sudan (mainly in the Western Equatoria State) and northern Uganda (the districts of Gulu, Kitgum, Pader and Lamwo), where it was also found to be associated with onchocerciasis [12,13]. Whether this syndrome is a separate entity or is part of the clinical spectrum of epilepsy associated with onchocerciasis has been a matter of debate for many years [14–16].

In this study, we investigated whether there was increased epilepsy prevalence in an endemic focus of onchocerciasis of the Orientale Province in the Democratic Republic of the Congo (DRC) and whether nodding syndrome was part of the clinical spectrum of epilepsy in this area. The study area was chosen because of medical observations reporting cases with nodding syndrome like clinical manifestations in two districts of the Orientale Province, namely the Ituri and the Bas-Uélé district. In the Ituri district, epilepsy was frequently reported in the medical history of young patients with onchocerciasis participating in a Moxidectin clinical trial conducted by WHO/TDR at the General Hospital of Reference Rethy between 2009 and 2012 [17]. In view of these observations, the Health Provincial Division of the Ituri district commissioned a scientific team to conduct an investigation of these cases of epilepsy. In February 2013, the investigation team led by MM visited the Logo health area in the Ituri District, and identified 6 cases with nodding syndrome like clinical manifestations [18]. In Bas-Uélé district, a team led by GMa and JK of the Congolese Ministry of Health investigated cases of “childhood epilepsy epidemic” in the Liguga health area where the reported *O*. *volvulus* nodules prevalence in 2012 was 66.7%, and identified also children with nodding syndrome like clinical manifestations. These observations led us to carry out a first exploratory mission to the Bas-Uélé district in the localities of Liguga, Dingila, and Titule in March 2014 followed by a second mission in June 2014 to Dingila and Titule. In this paper we describe the prevalence of epilepsy in Dingila and Titule. Moreover in Titule we examined potential risk factors for epilepsy, in particular the distance of each household to the major river.

## Materials and Methods

### Setting

**Fig 1:**
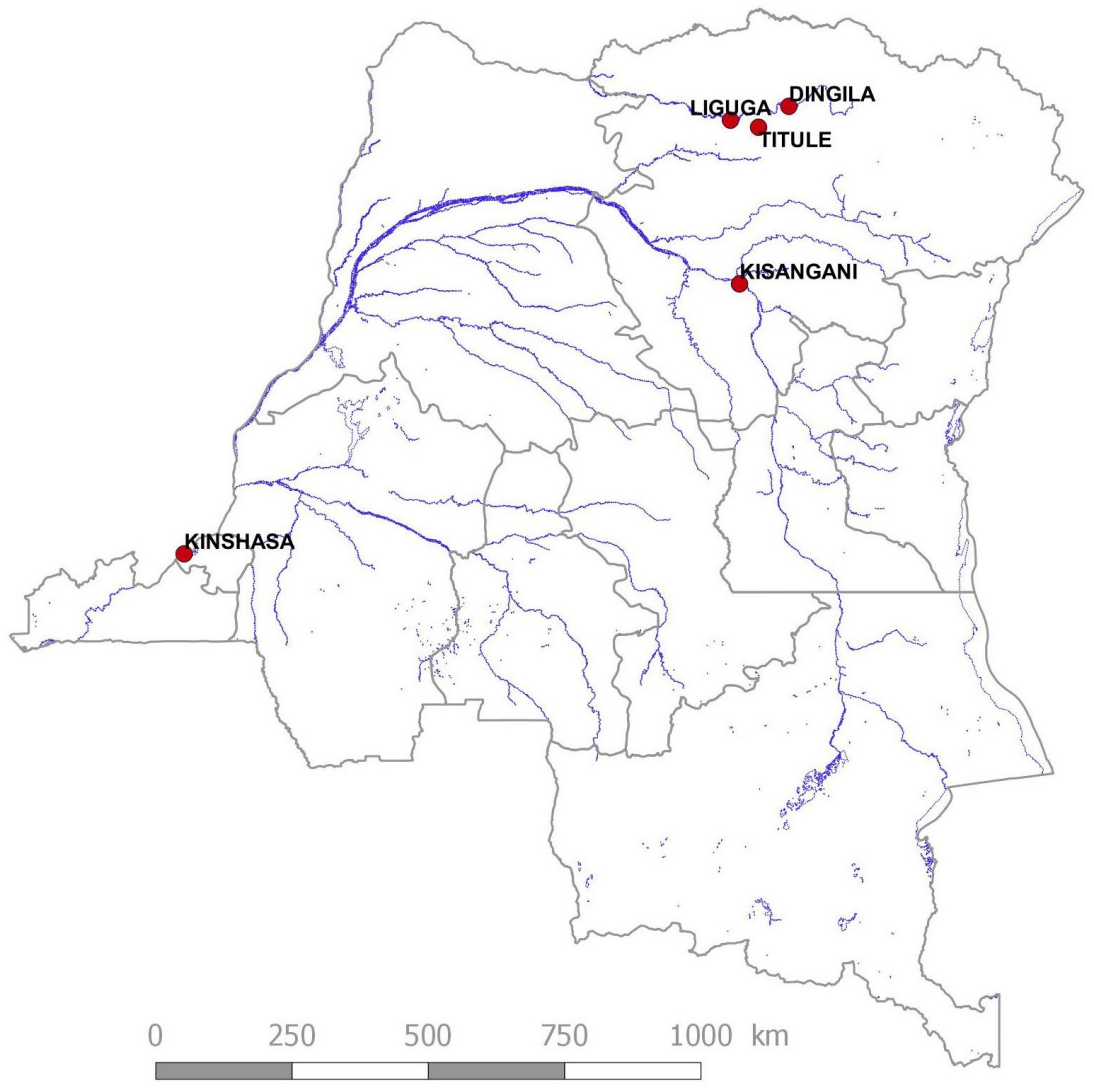
General location of the study sites.

Studies were performed in two localities of the Bas-Uélé district in the Orientale Province of the DRC, namely Dingila and Titule. The Bas-Uélé district has a tropical climate with a rainy season from April to November. Over the year, the average temperature in Dingila is 24.4°C. The average annual precipitation reaches 1,738 mm. January is the driest month of the year (34mm), while October is the wettest with an average rainfall of 255 mm. Vegetation cover consists mainly of dense tropical rainforest and riparian flora and bamboo patches in wetlands near major rivers, while savannah defines it in its most northern part along the border with the Central African Republic. Buta is the main town of the district, which is split into 11 health zones. The Titule health zone has a population of 73,432 inhabitants over an area of 5,450 km² with a density of 13 inhabitants/km². The Titule health zone is divided into 10 health areas among which Titule 1 and 2, were investigated. The main socio-economic activities are agriculture and fishing. Secondary activities include hunting, gathering, collecting snails, mushrooms, caterpillars and termites, or petty trade, while domestic poultry and small livestock are the main animal husbandry. The diet of the population is based mainly on the consumption of cassava and its derivatives, and plantain bananas. For many years, and especially in the period 1996–2002, the region experienced episodes of war which caused displacement of people into the surrounding forests. Titule is a locality crossed by the Bima River which is a major tributary and confluate with the Uélé in Liguga. Titule has a population of 11,882 inhabitants distributed between Titule 1: 4,776 (Gbulusu: 2,462, Mopemba: 2,314) and Titule 2: 9,220 (Basayo: 2,607, Mokoa: 3,896, Kpebele: 820). Dingila, was a prosperous city around the 1950’s where a major cotton wool farming company (the Compagnie de Développement du Nord, (CODENORD)) was located. Following two episodes of war, cotton production has been abandoned since 2001. The population of Dingila is estimated at 12,776 inhabitants. Both villages (Fig 1) are situated close to fast flowing rivers: Dingila (N3.68336—E26.03611) lies along the Uélé river and Titule (N03.28150 –E025.52894) along the Bima river.

### Definitions

A case of epilepsy was defined as a patient who lost consciousness at least twice with convulsions and without fever or any acute illness [19]. A case of suspected nodding syndrome was defined as a person who presented with episodes of decreased consciousness during which the head dropped forward repeatedly [20].

### Estimation of the epilepsy prevalence

In June 2014 families in Dingila with members known to have epilepsy were interviewed at home by two staff members of the ivermectin community distributors network (the *relais communautaires*). In total, 370 cases of epilepsy were recorded as well as information on age and sex of the epileptic patients, and age of the first epilepsy episode. The prevalence of epilepsy was determined using the total number of people living in Dingila as denominator.

In June 2014 a household survey was carried out in Titule by two physicians, FT and JK together with a nurse (FM) and two ivermectin community distributors. The team, split in two groups each led by a physician, visited every 3^rd^ household in four different directions of the village; the starting point was the Titule 1 health Centre. If household members were not at home, the next home was visited. All household heads and parents of children present in the household were interviewed in their local language. Families with and without epilepsy were geo-located (handheld Garmin 62Cs GPS; ±4m accuracy). For every consenting household, a one page questionnaire was completed (available as supporting information). Age and sex of every household member was recorded. Epilepsy is a condition that is well known by the local population; in the local language it is called “Epupuluga” and in lingala “Malali Ya Ndeke”. Interviews started by asking whether there was a family member with Epupuluga or Malali Ya Ndeke. Thereafter the doctors (FT and JK) asked the family members to describe the type of seizures (or to show what happens during a seizure), to report on the precipitant circumstances, the duration of seizures, whether they were associated with uncontrollable tongue biting or passing of urine or stool, whether there were family members with episodes of absence (sudden episodes of decreased consciousness of sudden onset and short duration) with or without nodding of the head, whether epilepsy had been treated and what the effect was of anti-epileptic treatment on seizures. Although the answers to these questions were not systematically recorded, the final doctors diagnosis was used in determining the prevalence of epilepsy.

For household members with epilepsy confirmed by the doctor, the age of the first epilepsy episode was noted. For those who developed epilepsy in 2014, the exact month of the first epilepsy attack. For every household member the question was asked whether ivermectin was taken in 2013 (the last year ivermectin was distributed at the time of the study).

### Spatial analysis

Of the 2906 cases in the original data file 2874 included valid geographic coordinates which were included in the subsequent spatial analysis. The spatial distribution of epilepsy cases over the study area was mapped by the location of their household of residence using a projected coordinate system. Clusters of increased epilepsy risk within the study area were located and mapped by applying a kernel density estimator to the geographical locations of households [21], weighted according to their respective prevalence rates. To test whether significant clustering of epilepsy prevalence exist and over what distance clustering occurs, Moran’s I statistic of spatial autocorrelation was calculated over incremental distance intervals of 50m starting at 200m. Before analysis, epilepsy prevalence rates per household were transformed using arcsine square root transformation for proportions.

Exposure to riverine habitat was determined as the Euclidian distance (in meters) between a household and the nearest flowing river. Since data on local river networks were not available, these were modelled based on a 30m SRTM digital elevation model, freely available online [22]. A model of the local hydrology was derived applying a triangular multiple flow direction algorithm [23] to the elevation data, from which a flow network was extracted. The resulting river network was validated using the point locations of rivers recorded in the field. The average distance between modeled river flows and river locations observed in the field was less than 30 meters, corresponding to the resolution of the original digital terrain model used. This observed accuracy was significantly less than the minimum distance between a household and the nearest river flow (Two sample t-test: t = 20.6, df = 151, p <0.001).

### Risk factor analysis

The association between the occurrence of epilepsy cases and past ivermectin treatment, and exposure to riverine habitat was determined by means of a Generalized Linear Mixed Model (GLMM) using a logistic link function [24]. Ivermectin use during the last year (in this survey, 2013) and the distance to the nearest river were entered as fixed effect into the model. To account for possible confounding effects due to gender and age, these were added as categorical fixed effects. To account for possible clustering of cases within a household, this was included in the model as a random effect.

### Ethics statement

The study was approved by the Institutional Review Board of the University of Kisangani. Written informed consent was obtained from all families who participated in the study.

## Results

### Dingila

Among the 12,776 individuals of Dingila, 373 (2,9%) individuals with epilepsy were identified; 193 (52%) females and 180 (48%) males; mean age: 18, IQR 13–22, range 2–62; mean age at onset of the epilepsy: 11, IQR 7–11, range 1–37.

**Fig 2:**
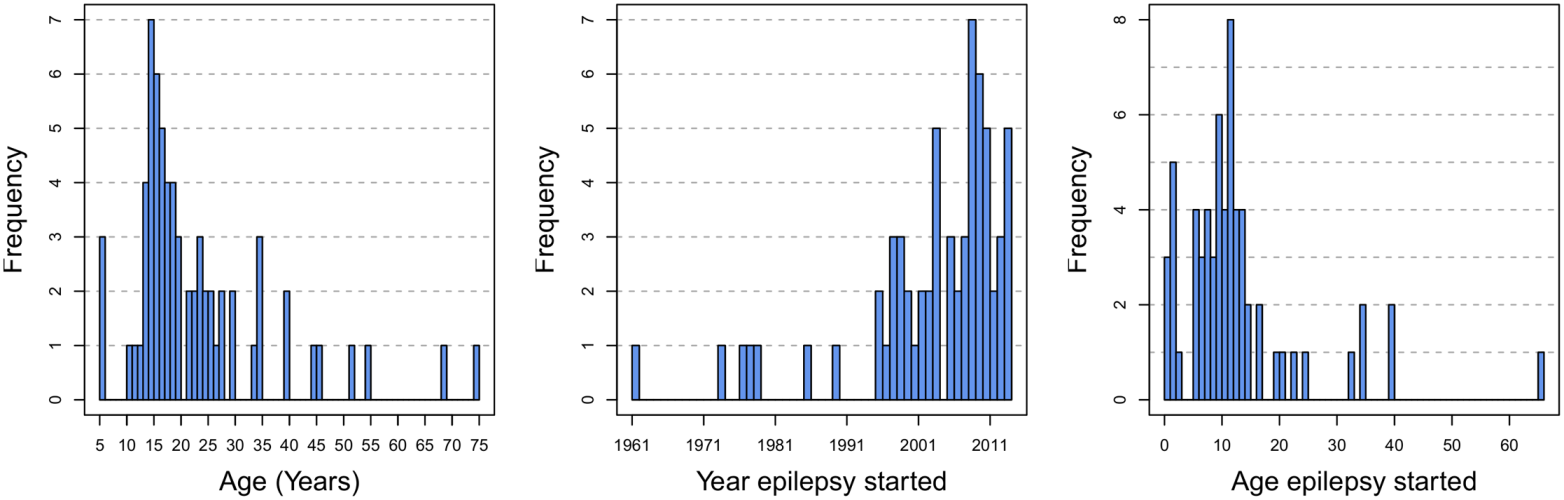
From left to right: the age distribution of epileptics in Dingila, the year their epilepsy started, and their age upon first epilepsy seizure.

The age distribution of these 373 individuals with epilepsy, and the year and age the epilepsy started are shown in Fig 2.

### Titule

**Fig 3:**
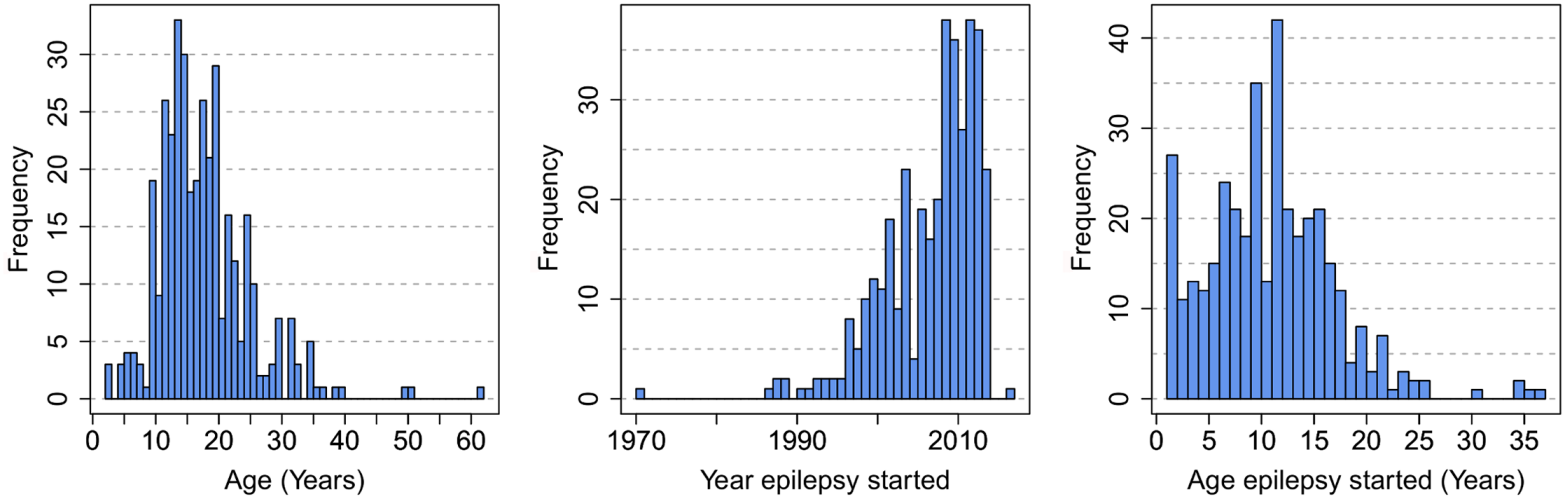
From left to right: the age distribution of epileptics in Titule, the year their epilepsy started, and their age upon first epilepsy seizure.

Three hundred and fifty one households of Titule I and II were surveyed over a period of 9 days; in total 2,906 people participated in the survey. Sixty seven (2.3%) reported episodes of epilepsy; 36 (53%) were male and 31 (46%) female with a median age of 19, range 5–75. In 3 (4%) of them head nodding was reported. In 6 families there were at least two persons with epilepsy and in one family three. All patients with epilepsy had seizures the last 5 years, and all except two during the last two years. The latter never took anti-epileptic treatment but received annual ivermectin treatment. Six individuals developed their first episode of epilepsy within the last 6 months (annual incidence = ±4.12/1000), 4 (67%) of them did not take ivermectin 7 months earlier. The age distribution of epilepsy cases, the year and age of onset of epilepsy are shown in Fig 3.

The highest prevalence of epilepsy was observed in the 10–19 age group (Table 1).

**Table 1:** Age-specific prevalence rates of epilepsy in Titule, Bas-Uélé District, Northern Congo.

Age (years)	Population of Household sample	N° of patients with epilepsy	Epilepsy prevalence (%)	For comparison: Age-specific rates from Western Uganda, 1994[25]
**> 40**	507	8	1.6	*0*.*6*
**30–39**	315	6	1.9	*1*.*8*
**20–29**	435	17	4.0	*1*.*7*
**10–19**	701	33	4.7	*8*.*3*
**< 10**	948	3	0.3	*1*.*4*
**All ages**	2906	67	2.3	*3*.*0*

### Spatial analysis

**Fig 4:**
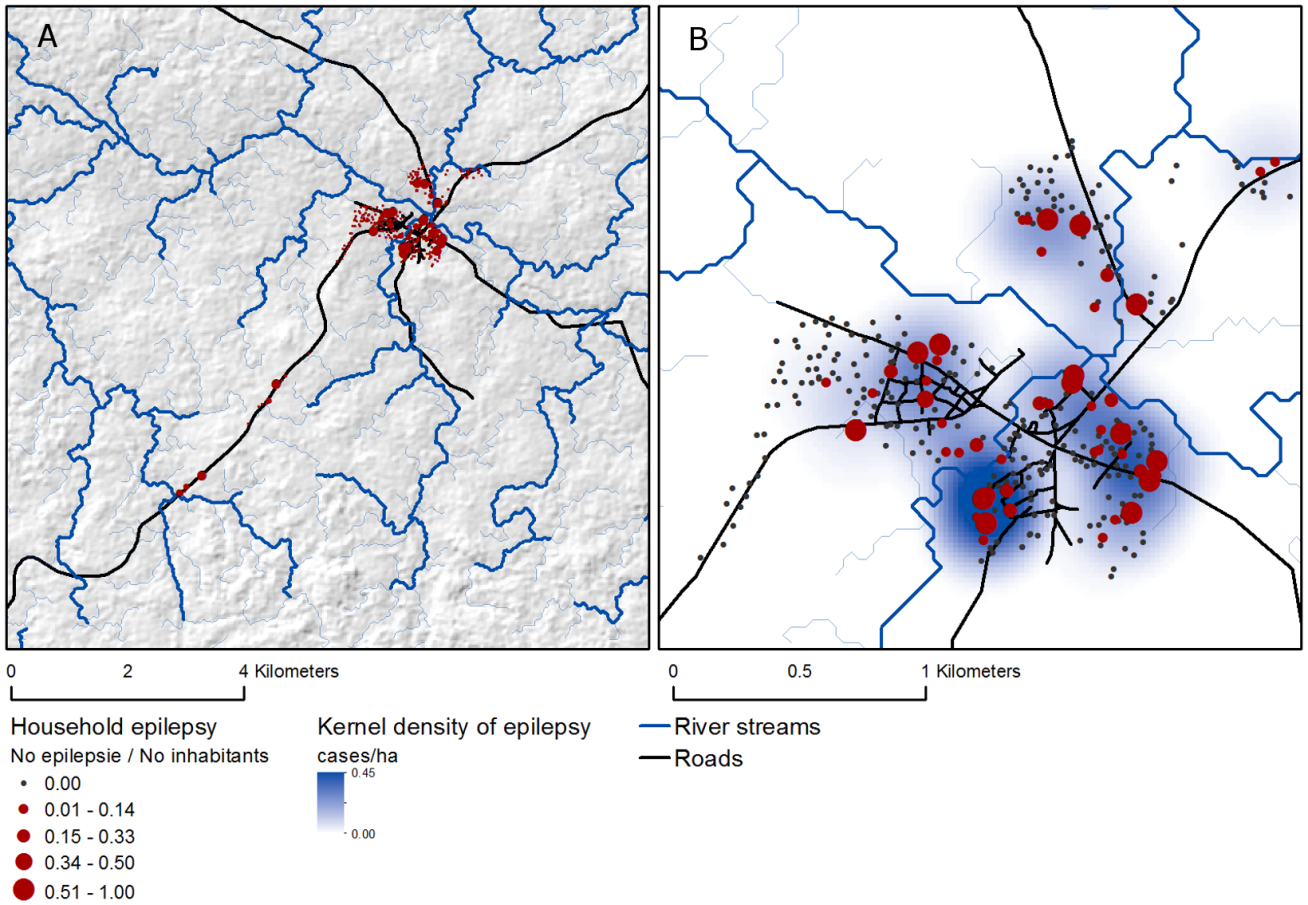
Overview of study area (a) and Titule village (b) showing the point locations and smooth kernel density estimation (300 m radius) of epilepsy in the area as well as the location of roads and rivers.

Mapping the number of epilepsy cases per household shows high heterogeneity in epilepsy across Titule village, indicative of spatial clustering of epilepsy cases (Fig 4).

**Fig 5:**
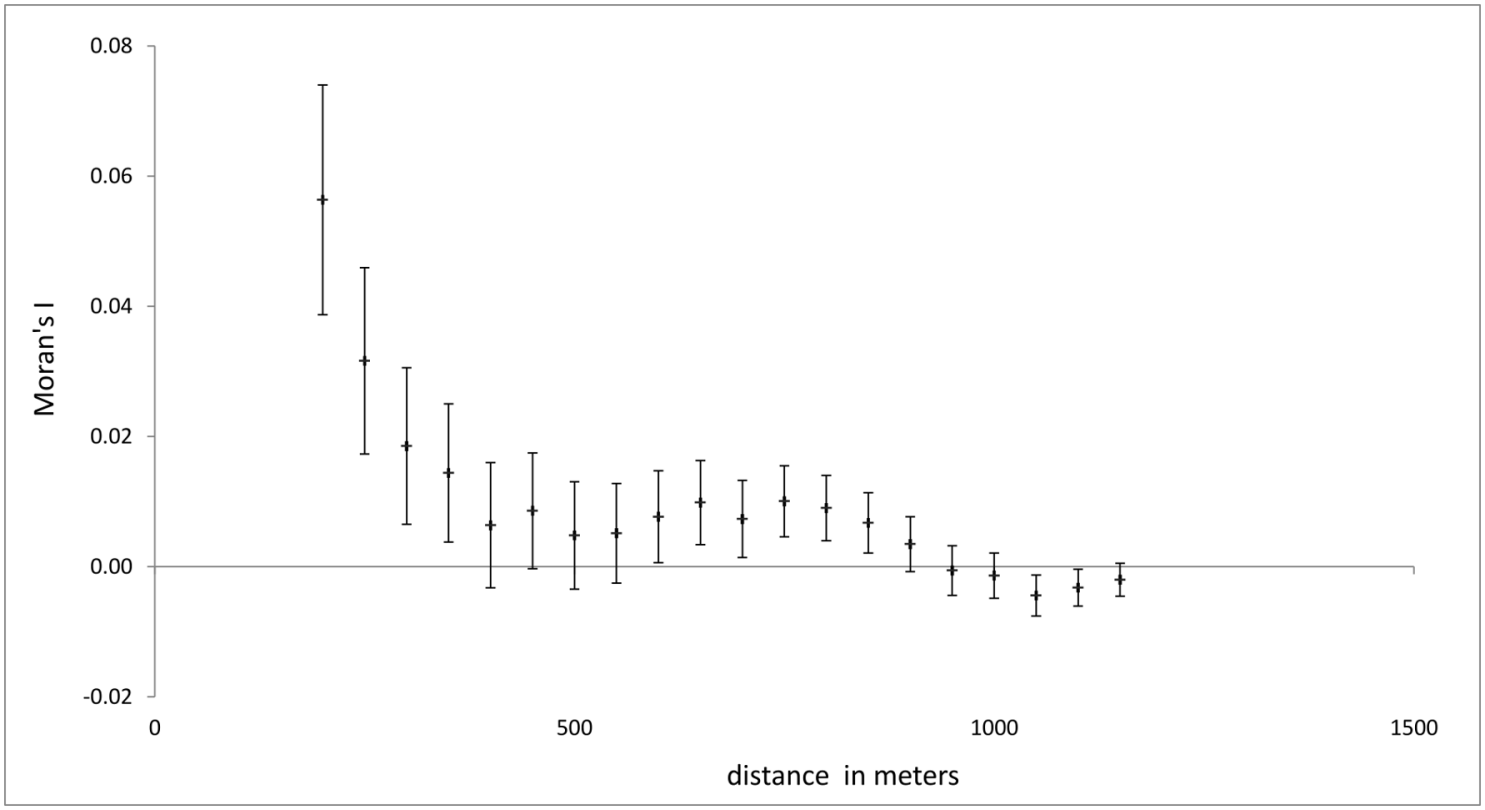
Plot showing the correlation between household prevalence rates as a function of distance (Moran’s I statistic of spatial autocorrelation). Spatial autocorrelation was calculated by incremental distance intervals of 50 meters starting at 200 meters.

From the spatial autocorrelation analysis over incremental distances it appears that the prevalence of epilepsy within households is significantly clustered at distances of 200–350 meters (Fig 5).

The kernel density estimate using a bandwidth of 300 meters, corresponding to the distance at which spatial clustering occurs, confirms the existence of several spatial clusters of increased epilepsy rates (Fig 4).

Table 2 shows the results of the multiple random effects logistic regression model relating the occurrence of individual epilepsy cases to the past usage of ivermectin, the position of a household relative to a river flow a person’s age (0–9, 10–19 and > 20 years of age) and gender. Clustering of cases within households was accounted for by adding households as a random effect to the model.

**Table 2:** Multivariate regression analysis of individual risk factors for epilepsy in Titule village Bas-Uélé District, Northern Congo.

Fixed effects:	Population in sample	No of patients with epilepsy	Epilepsy prevalence	OR^1^	(95%CI)^4^
**Intercept**	2874	66	2.3%	0.002	(0–0.006)***
**Ivermectine use**					
No	1594	48	3.0%	-	-
Yes	1280	18	1.4%	33.3	(0.12–0.44)***
**Age class**					
0–9	940	3	0.3%	1.33	-
10–19	693	33	4.7%	0.63	(9.8–113.2)***
> 20	1241	30	2.4%	0.002	(3.44–39.1)***
**Gender**					
Female	1508	35	2.3%	-	-
Male	1366	31	2.3%	33.3	(0.79–2.3)^ns^
**River distance**^2^				0.63	(0.45–0.91)*
**Household random effect**^**3**^				1.34	

^1^ Model intercept representing the baseline odds of epilepsy within the reference category at zero distance to a nearest river

^2^ River distances were centered on the mean (204 m) and scaled to the standard deviation (132 m).

^3^ Variance between random by-household intercept

^4^ p< 0.05 = *, p <0.01 = **, p <0.001 = ***, ns = not significant

A significant increase of epilepsy was observed in persons of 10–19 years of age (OR: 33.3, p<0.0001). Being treated with ivermectin over the preceding year resulted in lower odds of epilepsy (OR: 0.23; p < 0.001). Equally, living in a household at larger distance to a river reduced the risk of epilepsy (OR: 0.63; p < 0.001).

## Discussion

The prevalence of epilepsy in villages in Bas-Uélé district in the Orientale Province in the DRC was comparable with those reported in other onchocerciasis endemic areas in Africa [8] but higher than the epilepsy prevalence (0.4–1%) reported in most studies in the rest of the world [26–30]. In a study of 586,607 residents in five Health and Demographic Surveillance System (DSS) centers in sub-Saharan Africa, only 1,711 (0.29%) individuals were diagnosed as having active convulsive epilepsy [31]. Prevalence adjusted for attrition and sensitivity varied between sites: 7.8 per 1000 people (95% CI 7.5–8.2) in Kilifi, Kenya, 7.0 (6.2–7.4) in Agincourt, South Africa, 10.3 (9.5–11.1) in Iganga-Mayuge, Uganda, 14.8 (13.8–15.4) in Ifakara, Tanzania and 10.1 (9.5–10.7) in Kintampo, Ghana [28]. Interestingly, the highest prevalence of epilepsy was recorded in the Ifakara DSS in Tanzania, located not far from Mahenge, an onchocerciasis endemic region where nodding syndrome was first reported.

The peak incidence of epilepsy in patients in Dingila and Titule was around the age of 14–15. This is similar with other onchocerciasis endemic African regions and can most likely be explained by the equally high incidence of *O*. *volvulus* infection in these age groups and the cumulative nature of the *O*. *volvulus* infestation [9]. This peak incidence is in contrast with the epilepsy situation in industrialized countries and in non-onchocerciasis endemic regions in Africa where most onset of epilepsy is observed in the very young (< 5 years old) and in the older population [9]. In Cameroun, Pion et al., proposed to call this form of onchocerciasis associated epilepsy “river epilepsy” [9,20]. Although parents reported head nodding in three children, cases of nodding syndrome meeting the 2012 WHO case definition were not observed [20].

Important to note that in Titule, there was not only a high prevalence of epilepsy in the 10–19 age group (4.7%) but also in the 20–29 age group (4%). The latter is in contrast with the epilepsy prevalence reported in onchocerciasis endemic regions where ivermectin was not yet introduced. In a study from 1991 in Kyarusozi sub-county in western Uganda, 91% of the epilepsy cases were below the age of 19 [32]. In contrast, in the Imo river basin in Nigeria where ivermectin was distributed since 1994, in 2002, 67% of the patients with epilepsy were 20–29 years old [33]. This epilepsy age shift after the introduction of ivermectin is an argument that ivermectin may reduce the incidence of epilepsy.

Our study has several limitations. In most individuals the epilepsy was only reported and not observed. Individuals with epilepsy were not examined by a neurologist and laboratory investigations and Magnetic Resonance Imaging were not performed. In Dingila, only households known to have members with epilepsy were visited. Families who have family members with frequent generalized tonic–clonic seizures are generally known in villages by the *relais communautaires* who know their communities as they are in frequent contact with families and visit all households annually for the distribution of ivermectin, vaccination campaigns and mosquito net distribution. Nevertheless, it is possible that we underestimated the prevalence in Dingila, because individuals with other forms of epilepsy and with infrequent seizures may have been missed. Moreover in both villages we did not use a validated epilepsy questionnaire as was used in other studies in the past [34]. In Titule, we did not use the recommended two or three-stages standard screening methodology for epilepsy prevalence studies [34]. We conducted the survey differently due to time constraint, and because we had the possibility that 2 medical doctors could screen for and diagnose epilepsy directly in households, located not too far from each other. In our definition of epilepsy we did not specify a time limit for laps between the 2 first seizures, or for the last seizure and we only included convulsive epilepsy. Therefore comparison of our results with published epilepsy prevalence data is difficult. In Dingila, as the diagnosis of convulsive epilepsy was not confirmed by a medical doctor, it is possible that certain other conditions such as psychogenic non-epileptic seizures were misdiagnosed as epilepsy. Moreover to determine the prevalence of epilepsy in Dingila we used a census figure as denominator, that was not verified at the moment of the study by a house-to-house survey.

Initially we expected that cysticercosis was a possible cause of epilepsy in the region because many pigs were observed in the villages and because cysticercosis is known to be an important cause of epilepsy in Africa [35]. However in a case control study performed in Titule, none of the cases or controls had *Taenia solium* antibodies, suggesting that cysticercosis was not an important cause of epilepsy in the region [36]. In Titule, both onchocerciasis and *Loa Loa* are endemic and ivermectin distribution is given to the population regardless whether a person has a *Loa Loa* infection. Indeed, following the administration of ivermectin in *Loa Loa* infected patients, with high filarial loads, there is a higher risk (1/10 000) of developing serious adverse effects mainly caused by the massive death of *Loa Loa* filariae in the brain causing an encephalopathy and eventually coma and death [37]. However of the 59 cases and 61 controls in our case control study in Titule only one case and one control had a positive *Loa Loa* PCR blood test [36]. Therefore *Loa Loa* co-infection cannot explain the high prevalence of epilepsy in the region. On the other hand, in 78% of cases and 83% of controls *O*. *volvulus* IgG4 antibodies were found [36].

Individuals who used ivermectin in 2013 were less likely to have epilepsy compared to those who did not use ivermectin in 2013. The explanation for this could be that certain individuals in Titule with epilepsy also in the past were not taking ivermectin and therefore were not protected against onchocerciasis and onchocerciasis associated epilepsy, or that people with epilepsy avoid taking ivermectin because they may suspect ivermectin is causing epilepsy. Two (33%) of the 6 patients who developed epilepsy during the last 6 months had taken ivermectin 7 months before. If this information is true, this suggest that if ivermectin offers some protection against onchocerciasis associated epilepsy, this protection may not be complete with only one dose of ivermectin per year.

The distribution of epilepsy cases within Titule showed a marked spatial pattern. Epilepsy were observed to cluster both within and between adjacent households located near river flows. A possible explanation for this observed pattern could be the fact that households near river flows are more often exposed to *O*. *volvulus* vectors (*Simulium sp*.) which occupy riverine habitats, resulting in increased infestation rates.

In conclusion, we documented a high prevalence of epilepsy in the Bas-Uélé district, in villages close to rapid flowing rivers infested by blackflies. Identifying whether an *O*. *volvulus* infestation can directly or indirectly cause epilepsy, or whether there could be another agent transmitted by blackflies responsible for the epilepsy require further investigation [16,38].

## Supporting Information

S1 Checklist STROBE Checklist. (DOCX)

S1 Text Questionnaire used for the prevalence survey. (DOCX)
