# Supplementary material for: Prevalence of River Epilepsy in the Orientale Province in the Democratic Republic of the Congo
Source: PLoS Negl Trop Dis. 2016 May 3;10(5):e0004478. doi: 10.1371/journal.pntd.0004478 (PMC4854481; doi:10.1371/journal.pntd.0004478)
Supplement: S1 Text — (DOCX) [file pntd.0004478.s002.docx]

| **No. of people in household** | **Actual age** | **Gender** | **Epilepsy**  **(unprovoked loss of consciousness with generalized convulsions, no fever)** | **Other form of epilepsy,nodding?** | **At least 2 seizure episodes** | **Age when epilepsy started** | **If started in 2014-15 how many months ago?** |  |  |  |  |  |  |  |  |  |  |  |  |  |  |  |  |
| --- | --- | --- | --- | --- | --- | --- | --- | --- | --- | --- | --- | --- | --- | --- | --- | --- | --- | --- | --- | --- | --- | --- | --- |
|  |  |  |  |  |  |  |  | 2014 | 2013 | 2012 | 2011 | 2010 | 2009 | 2008 | 2007 | 2006 | 2005 | 2004 | 2003 | 2002 | 2001 | 2000 | 1999 |
| **1** |  | **M F** |  |  |  |  |  |  |  |  |  |  |  |  |  |  |  |  |  |  |  |  |  |
| **2** |  | **M F** |  |  |  |  |  |  |  |  |  |  |  |  |  |  |  |  |  |  |  |  |  |
| **3** |  | **M F** |  |  |  |  |  |  |  |  |  |  |  |  |  |  |  |  |  |  |  |  |  |
| **4** |  | **M F** |  |  |  |  |  |  |  |  |  |  |  |  |  |  |  |  |  |  |  |  |  |
| **5** |  | **M F** |  |  |  |  |  |  |  |  |  |  |  |  |  |  |  |  |  |  |  |  |  |
| **6** |  | **M F** |  |  |  |  |  |  |  |  |  |  |  |  |  |  |  |  |  |  |  |  |  |
| **7** |  | **M F** |  |  |  |  |  |  |  |  |  |  |  |  |  |  |  |  |  |  |  |  |  |
| **8** |  | **M F** |  |  |  |  |  |  |  |  |  |  |  |  |  |  |  |  |  |  |  |  |  |
| **9** |  | **M F** |  |  |  |  |  |  |  |  |  |  |  |  |  |  |  |  |  |  |  |  |  |
| **10** |  | **M F** |  |  |  |  |  |  |  |  |  |  |  |  |  |  |  |  |  |  |  |  |  |
| **11** |  | **M F** |  |  |  |  |  |  |  |  |  |  |  |  |  |  |  |  |  |  |  |  |  |
| **12** |  | **M F** |  |  |  |  |  |  |  |  |  |  |  |  |  |  |  |  |  |  |  |  |  |
| **13** |  | **M F** |  |  |  |  |  |  |  |  |  |  |  |  |  |  |  |  |  |  |  |  |  |
| **14** |  | **M F** |  |  |  |  |  |  |  |  |  |  |  |  |  |  |  |  |  |  |  |  |  |
| **15** |  | **M F** |  |  |  |  |  |  |  |  |  |  |  |  |  |  |  |  |  |  |  |  |  |
| **16** |  | **M F** |  |  |  |  |  |  |  |  |  |  |  |  |  |  |  |  |  |  |  |  |  |
| **17** |  | **M F** |  |  |  |  |  |  |  |  |  |  |  |  |  |  |  |  |  |  |  |  |  |
| **18** |  | **M F** |  |  |  |  |  |  |  |  |  |  |  |  |  |  |  |  |  |  |  |  |  |
| **19** |  | **M F** |  |  |  |  |  |  |  |  |  |  |  |  |  |  |  |  |  |  |  |  |  |
| **20** |  | **M F** |  |  |  |  |  |  |  |  |  |  |  |  |  |  |  |  |  |  |  |  |  |

Preliminary Questionnaire for Cross-sectional study (HOUSEHOLD)

Country: __________________ Region: __________________ District: __________________

Ward/County: __________________ Village/Parish: __________________

Village leader’s name: __________________ Household(HH) no.: __________________
